# Life imitating art: Depictions of the hidden curriculum in medical television programs

**DOI:** 10.1186/s12909-015-0437-8

**Published:** 2015-09-26

**Authors:** Agatha Stanek, Chantalle Clarkin, M Dylan Bould, Hilary Writer, Asif Doja

**Affiliations:** 1 Faculty of Medicine, University of Ottawa, 451 Smyth Rd, Ottawa, ON K1H 8M5 Canada; 2 Children’s Hospital of Eastern Ontario, 401 Smyth Rd, Ottawa, ON K1H 8L1 Canada

**Keywords:** Education, Medical, Hidden curriculum, Television

## Abstract

**Background:**

The hidden curriculum represents influences occurring within the culture of medicine that indirectly alter medical professionals’ interactions, beliefs and clinical practices throughout their training. One approach to increase medical student awareness of the hidden curriculum is to provide them with readily available examples of how it is enacted in medicine; as such the purpose of this study was to examine depictions of the hidden curriculum in popular medical television programs.

**Methods:**

One full season of *ER, Grey’s Anatomy* and *Scrubs* were selected for review. A summative content analysis was performed to ascertain the presence of depictions of the hidden curriculum, as well as to record the type, frequency and quality of examples. A second reviewer also viewed a random selection of episodes from each series to establish coding reliability.

**Results:**

The most prevalent themes across all television programs were: the hierarchical nature of medicine; challenges during transitional stages in medicine; the importance of role modeling; patient dehumanization; faking or overstating one’s capabilities; unprofessionalism; the loss of idealism; and difficulties with work-life balance.

**Conclusions:**

The hidden curriculum is frequently depicted in popular medical television shows. These examples of the hidden curriculum could serve as a valuable teaching resource in undergraduate medical programs.

**Electronic supplementary material:**

The online version of this article (doi:10.1186/s12909-015-0437-8) contains supplementary material, which is available to authorized users.

## Background

The hidden curriculum is a set of influences that function at the level of organizational structure and culture [1–6]. It is comprised of processes, pressures and constraints which fall outside the formal curriculum, and are often unarticulated or unexplored [2]. In essence, the hidden curriculum represents what an institution teaches without intending or being aware it is taught. The realities of the hidden curriculum are dynamic in nature and shaped by not only structural processes but also by the ability of medical educators to consider and recognize their existence [6]. This differs from the informal curriculum, which takes place in interpersonal forms of teaching, such as among medical faculty and students. Such teaching is typically unscripted and *ad hoc* in nature [1–6]. Both the hidden curriculum and informal curriculum however, suggest the existence of a discrepancy between traditional forms of teaching and what the student retains.

All physicians and future physicians receive education through formal curricula delivered at both the undergraduate and postgraduate levels of medical training. The hidden curriculum can be considered to be ever present among medical students and residents [7] and, as argued by Hafferty, the hidden curriculum may impact medical trainees more than the formal curriculum itself, [8, 9]. It has been demonstrated to exert a wide influence on students, such as when choosing their specialty or when deciding whether to disclose personal errors in surgery [10–14].

The hidden curriculum can be observed in the *cultures*, *processes* and *structures* inherent in the practice of medicine. An example of *cultures* in medicine would be the unspoken hierarchy regarding the manner in which trainees are quizzed on inpatient rounds. In North America, usually the easier questions are targeted towards junior trainees, whereas more difficult questions are addressed towards more senior trainees. In North America, the “rule” which must be learnt is that it is considered improper for a more junior trainee to answer a question directed at a more senior trainee. *Processes* refer the manner in which the daily practice of medicine is carried out. For example, medical students are often told to spend significant amounts of time with patients to obtain not only the medical history, but to pursue the patient’s perceptions of illness and the impact disease has had on their lives. However, when they get to the clinical setting, trainees sometimes find they are told they are taking too long, and need to improve their efficiency in a busy outpatient clinic or the emergency room. *Structures* can be thought of as the way in which larger organizations govern medical practice. An example of these would be the physical layout of some emergency rooms, which is often dictated by hospital budgets. Students are often surprised to find that while patient privacy is stressed in medical school, often the only thing separating two patients in the emergency room is a thin curtain, which significantly limits how “private” a physician-patient conversation can be in that environment.

At a societal level, medical television programs can act as educational agents, and have demonstrated the ability to shape patient-physician communication [15]. They can also influence patient expectations of their physicians and resulting satisfaction [16, 17]. Literature attests to the potential of medical television dramas and reality programs to provide health information regarding medical diseases for its viewers [18]. More recently, studies suggest medical television may also serve as a tool in educating physicians in training [19]. Hirt and colleagues formulated a guide that summarizes eight popular television dramas and their specific potential for implementation in medical education [19]. There are, however, no studies that consider the depiction of the hidden curriculum in medical television or the impact these representations may have on developing medical trainees. As such, the objective of this study was to examine the depiction of the hidden curriculum in popular medial television programs. These depictions could potentially serve as a learning tool to increase trainee awareness of the hidden curriculum, with readily available vignettes.

## Methods

### Selection of television programs

Three television programs were chosen for the study: the highest-rated medical drama from the 1990’s (*ER*), the highest-rated medical drama from the 2000’s (*Grey’s Anatomy*) and the highest-rated medical comedy from the 2000’s (*Scrubs*) (http://tviv.org/Nielsen_Ratings/Historic/Network_Television_by_Season/2000s) [20–22]. One entire season from mid-way through the series’ was viewed (i.e., Season 7 for *ER*, Season 5 for *Grey’s Anatomy*, and Season 4 for *Scrubs*) [20–22]. This selection of season was justified as most representative as television programs often establish the plot and develop the characters in their first few seasons and focus on storyline resolution in their final years.

### Viewing protocol

All episodes of each respective season for the three medical dramas were viewed by one investigator (AS). 22 episodes of ER, 24 episodes of *Grey’s Anatomy*, and 24 episodes of *Scrubs* were watched, for a total of 70 episodes. Notes were taken at 10-min time intervals to ensure a minimum consistent amount of note taking. This involved a brief interruption in viewing to document aspects of each episode such as plot lines, character development, and general tone, which allowed for a greater contextual understanding of the abstracted exemplars of the hidden curriculum.

### Data collection and analysis

We conducted a summative content analysis that was both inductive and deductive in nature, to identify depictions of the hidden and informal curriculum in the television medical programs [23]. A preliminary inductive glossary of terms relating to the hidden curriculum was developed by content experts based on the literature, and served as the initial coding scheme for data abstraction (Additional file 1) [1, 4, 13, 14, 19]. Throughout data collection, the coding scheme was revised to reflect emergent themes derived from the medical dramas. Specific examples of the hidden and informal curriculum were abstracted, documented and described in detail in a spreadsheet. For each occurrence of the hidden curriculum, the following data were recorded: episode number, scene length and timing, and major themes or conflicts being demonstrated in the scene (Additional file 1). This process allowed us to systematically condense a large volume of raw television data into categories and latent themes based on interpretation [24]. To enhance the trustworthiness of the findings, a second reviewer (CC) independently reviewed a subsample of episodes selected at random from each series (*n* = 6). These episodes were reviewed using the established coding scheme and procedures. The two reviewers then met to discuss their initial interpretations and coding, and areas of divergent interpretation were discussed at length until agreement was reached.

This study was performed solely by examining television programs. As such, this research does not involve human subjects, human material or human patient data; for this reason, ethical approval from our institutional research ethics board was deferred.

## Results

Exemplars of the hidden curriculum were tabulated and subjectively rated as poor, moderate, or excellent to reflect the quality of the depiction of the hidden curriculum. These ratings were developed by the authors in terms of their ability to serve as teaching tool vignettes. *Excellent exemplars* demonstrated key aspects of the hidden curriculum and did not require an understanding of character or plotline. As an example, in episode 7 of *Grey’s Anatomy*, a senior staff physician witnessed three interns arguing over the care of an unstable patient for their respective personal gains and openly criticizes them for “acting like vultures”. He expresses his disappointment and disdain at their subpar respect and lack of concern for the patient’s well-being, indicating a very clear example of patient dehumanization. *Moderate exemplars* required some additional context for understanding. For example, in episode 22 in *Grey’s Anatomy*, an intern speaks to a junior member of her team in a condescending manner, indicating an unacceptable example of role modeling. However this example requires additional viewing time of the show for the viewer to appreciate the personal issues affecting the intern’s performance at work. *Poor exemplars* featured nuanced enactments of the hidden curriculum that were character or plot driven. As an example, in episode 10 of *Scrubs*, a senior physician pages any intern who is available to assess a distinguished member of the community, with a resulting overly dramatic depiction of the entire team of interns rushing to get to the appropriate locale first.

*Grey’s Anatomy* had a recorded 39 episodes of the hidden curriculum with no poor, 13 moderate, and 26 excellent examples. *ER* also had no poor examples of the hidden curriculum, with 2 and 27 moderate and excellent examples, respectively, out of a total of 29 recorded examples. Finally, *Scrubs* totaled 25 examples with 2 poor, 8 moderate, and 15 excellent recorded vignettes.

The hidden curriculum was found to be enacted most frequently in five ways across the medical dramas. These depictions, reported as themes, included: *the hierarchical nature of medicine*, *unprofessionalism*, *patient dehumanization*, *challenges to work-life balance*, and *role modeling*. We also found 3 minor themes that were demonstrated in only a few shows: *consequences of the hidden curriculum*, *staging* and *faking it* (Table 1). For our analysis, we focused on the five most prevalent major themes. Table 2 summarizes the frequency of occurrence of these five themes, and they are described in more detail below (see also Additional File 1):

**Table 1:** Major and minor themes of the hidden curriculum as depicted in television dramas

Theme	Definition	Example
Major themes		
Hierarchical nature of medicine		
*Discipline*	This centers around the primacy put on certain specialties and sub specialties.	Perceived “superiority” of some specialties over others
*Position in the hierarchy*	The reinforcement of junior-senior interprofessional relations and positions within the medical hierarchy.	A staff physician belittling a junior resident
*Patient perspective*	Patients reinforcing hierarchy among medical trainees.	Patient refuses the services of a younger-looking professional over an older professional
Unprofessionalism	This refers to an individual’s behavior that demonstrates a lack of integrity or responsibility from others	Unprofessionalism from a staff physician
		Evidence of tolerance of unprofessional behavior
Patient dehumanization	A broad term that demonstrates a lack of empathy or respect for patients from medical professionals.	Treating patients as objects/ data sources
		Not referring to patients by name but by diagnosis
Life balance	This encompasses the challenges experienced by all medical professionals in keeping an equilibrium between hours worked and the demands of personal/ family life.	Putting aside personal time to stay beyond the time needed for a shift
Role modeling	How medical students “model” exemplary behavior of staff doctors	Trainees modeling/ tailoring behaviors to meet needs of staff doctors
		Discussions regarding behaviors they ‘have to model’ but will not continue when they are independent
Minor themes		
Consequences of the hidden curriculum	This refers to the loss of idealism among medical students as they progress from their first year into their residency and beyond.	Loss of idealism of trainees
		Emotional neutralization/ suppression
		Adoption of a “ritualized” professional identity
		Change in ethical integrity
		Shift from focus on patient care to medical technology or nature of procedure
Staging	Challenges during transitional stages in medicine	Discrepancy between what is seen in theory and on the wards
Faking it	Reflects dishonest behavior for personal gain regarding prestige, competition, and favoritism.	Overstating capabilities

**Table 2:** The frequency of occurrence of the five most prevalent themes of the hidden curriculum as depicted in television dramas

	Television show		
Theme	*Grey’s Anatomy*	*ER*	*Scrubs*
Hierarchical Nature of Medicine	19	10	10
Unprofessionalism	7	8	8
Patient dehumanization	6	4	3
Life balance	2	4	0
Role modeling	1	4	0

### The Hierarchical nature of medicine

*The hierarchical nature of medicine* was the most frequent depiction of the hidden curriculum; the concept of a pyramidal system of superiority was common to all of the medical dramas. In Table 2, hierarchy is further subdivided by position in the medical field, specialty, and patient point of view. An example of the hierarchy that occurs among differing subspecialties is portrayed in episode 6 of *Grey’s Anatomy*. In this scene a plastic surgeon is appalled to see a poorly performed closure of a facial injury by his new colleague, a trauma surgeon. The resident working with the trauma surgeon justifies the nature of the closure due to the acute situation of the patient. The plastic surgeon is nonetheless unimpressed and does not agree with the approach. He consequently comments and laughs with a nearby neurosurgeon, in front of the resident: “trauma guys- they just slap it together*.”* While such a comment may be viewed as comedic and harmless for viewers, it also reinforces the perception of one surgical specialty being superior to another.

### Unprofessionalism

*Unprofessionalism* was the second-most frequent example of the hidden curriculum among the three viewed medical television dramas. *Unprofessionalism* was noted in scenes that promoted a sense of complacency regarding professional standards and behaviors, and during times of personal and professional conflicts. *Unprofessionalism* included the demonstrated lack of respect to patients and staff exhibited by all members of the health care team, such as staff physicians, medical students and residents, as well as nurses, social workers, or billing agents. In episode 16 of ER, a female surgeon experiences unprofessional conduct. In this exemplary scene, a heavily pregnant surgeon in an advanced stage of pregnancy is harassed by her senior colleague, regarding her physiological state and capacity to perform in the operative theater. The male surgeon openly questions whether she is still able to work. Her confident reply indicating that she is indeed more than capable is faced with skepticism. In fact, her colleague expresses his doubts regarding her ability “*to reach the operating table.”* Such instances reinforce the presence of unprofessionalism in medicine. This particular example also touches on the inequality females may experience in the field of surgery, a historically male-dominated field.

### Patient dehumanization

*Patient dehumanization* encompasses impersonal medical routines surrounding patient care that lack dignity, empathy, or privacy. *Patient dehumanization* is commonly noted at times when patients are vulnerable, in cases of sedation, confusion, or awaiting an invasive procedure. When multiple members of a health team are discussing a patient’s case without acknowledging that patient’s verbal or nonverbal behavior, this theme also comes to light. An example of patient dehumanization is observed in episode two of *ER*, when a nurse notices a father standing outside a glass door while a medical team is engrossed in the examination of his dying infant. Due to a lack of explanation or education on behalf of the team, the father believes that the teaching session and exam are a therapeutic intervention for his child. Realizing this, the nurse intervenes during the physical exam and advocates for the rights of the patient and family. She speaks with the senior physician to request that the medical team stop treating the infant “like a science experiment” while the father is present. The nurse is appalled that the team has not approached the parent to explain their assessments and procedures, and have failed to treat the dying infant with dignity.

### Work-life balance

Issues relating to difficulties establishing and maintaining a sense of *work-life balance* are readily depicted in both *Grey’s Anatomy* and *ER*, but are not featured in the viewed season of *Scrubs*. The challenge experienced by medical personnel to find a balance between personal commitments and their professional responsibilities is highlighted during times of promotion or transitional stages in careers. The tensions between professional and personal commitments are also emphasized during special moments in personal lives or times of increased family responsibilities, for example, the care of young children. In episode 1 of *ER*, an emergency room physician is offered a promotion by his superior. His superior makes it clear, however, that part of the job description will involve extreme dedication and, “no excuses,” in reference to personal relationships or obligations. This interaction infers that in order to achieve career success one must privilege work commitments over all others, including health, family and leisure. This point resonates with the challenges associated in finding equilibrium between professional and personal responsibilities and suggests success is merely a reflection of professional advancements.

### Role modeling

*Role modeling* can be portrayed both positively and negatively. In episode three of *Grey’s Anatomy*, a junior resident is presented with a highly sought-after opportunity to scrub in for a rare surgical case, under the supervision of a notoriously demanding plastic surgeon, who had paid little attention to her up until that point. Prior to this opportunity, the resident conducted meticulous research surrounding the medical case, ultimately leading the team to the correct diagnosis. When the resident politely declines the offer for personal reasons, she is informed by the plastic surgeon that “there will be no next time”. The staff is abrupt in his manner and limits further interactions with the intern. This scenario reflects negative role modeling by the staff surgeon, who has limited interest and patience for the needs of his student learners. While it may be appropriate to show disappointment, the resident should not be punished for this refusal, with respect to future exciting medical opportunities. A loss of opportunity to learn may hinder the resident’s training and later competencies as a potential surgeon. The scene concludes with the trainee feeling torn and disappointed.

## Discussion

### Fictional depictions of the hidden curriculum vs reality

Fictional depictions of the hidden curriculum identified in television programs reflect real-life examples of the hidden curriculum. While we did not rate exemplars based on our interpretation of how realistic they were, our findings suggest that enactments of the hidden curriculum in medical dramas may reflect some healthcare realities. All themes established in this study overlap with those in previous literature examining real-life examples of the hidden curriculum among medical trainees. The themes of *hierarchy*, *personal versus professional life balance*, *‘faking it’*, *staging*, *the competitive nature of medicine*, and *role modeling* were readily identified in many studies [12, 13, 25].

Interestingly, there were certain themes described in other studies of the hidden curriculum that overlapped with those in our study; we labeled these themes differently than the other studies, but the themes reflected similar content. For instance, our theme of *consequences of the hidden curriculum*, (Table 2) includes the suppression of normal emotional responses and emerging accountability that are considered by Gaufberg and colleagues as independent themes [13]. Furthermore, the theme of *personal encouragement*, as identified by Lempp and Seale’s work, was defined as the presence of positive role models and their ability to motivate students [25]. We categorized such instances in the television programs as a reflection of *role modeling*,; however, we found few positive examples of role modeling with staff and surgical interns in various stages of training. This may reflect the heightened reality of television or an effort to garner viewer interest by emphasizing negative behavior by role models. *Variable respect for patients*, a theme identified by Lamiani, described certain physicians who lacked respect for their patients and others who were kind and engaging [12]. For the purpose of our study, we categorized such instances as either examples of unprofessionalism or role modeling. *Disease-centered medicine* was another theme by Lamiani that overlapped with our study’s theme of *patient dehumanization*. We noted many scenes which depicted patient-physician relations, with a primary focus on a patient’s disease and not on the patient as a whole and classified such vignettes as reflections of *patient dehumanization*. Differing semantics, which may reflect the subjective process of identifying themes of the hidden curriculum, may account for such discrepancies. Despite these differences, there is substantial overlap of content of the hidden curriculum among television programs and actual medical trainee experiences.

*Protecting patients* was used to denote the approach of optimism for patient conditions and the need to calm patients by Lamiani [12]. We collected examples of similar examples in which physicians limited information or did not disclose the truth completely to patients, but labeled these instances as examples of *unprofessionalism*. Lamiani discusses that the paternalistic nature of the Italian health care system may explain why a theme may have a positive or negative connotation, depending on the cultural milieu [12].

### Differing depictions of the hidden curriculum in medicine

There are several themes of the hidden curriculum discussed in the literature, which were not identified among television programs in this study. These themes included: *positive experiences of human connection; haphazard teaching*; *the power differential* and a *delegation of patient’s emotional needs to nurses* [12, 13, 25]. *Human connection* is a general term for situations that encourage the formation of longitudinal relationships among medical personnel, through learning names, celebrating together, and transitions [13]. This theme was not identified among the three television programs. Another theme we did not identify was *haphazard teaching*, which was described by Lempp and Seale as the tendency for clinical staff to disregard timetables for teaching classes [25]. This particular study examined concepts of the hidden curriculum among real–life student experiences in the classroom. In contrast, all three television programs we viewed were set in an institutional clinical setting among medical staff and students, who were beyond this level of training. As such, we believe that this difference in learning environments plays a major role in this discrepancy [12]. *Power differential* – defined as the gap in approachability between physicians and patients – was also not noted in this study [12]. This theme encompassed instances in which physicians used jargon with patients or in which patients showed submissiveness with senior physicians and not medical students.

Finally, *delegating emotional patient needs to nurses* was also not identified in our study. As the television programs we viewed centered on physicians and residents, with limited screen time with other health care professionals, we believe that this accounts for this difference.

### Unique depictions of the hidden curriculum in medical television programs

We identified several unique themes of the hidden curriculum particular to television programs, which have not been well documented in studies on real life examples of the hidden curriculum. For example, *unprofessionalism* included depictions of physicians demonstrating a lack of respect for their patients; however we also identified instances of junior trainees not only experiencing unprofessionalism, but also tolerating the phenomenon itself. In addition, while the literature attests to the phenomenon of hierarchy in medicine, we specifically identified the issue of *hierarchy* in terms of a patient’s perspective, specifically how patients prefer to be treated by staff physicians as opposed to physicians-in-training. This may have been noted in our study because as the viewer, we are able to observe different viewpoints during the full patient and physician encounter. Most studies on the hidden curriculum in medicine only focus on interviewing medical professionals and therefore do not examine the perspective of patients.

### Comparing and contrasting educational values of various programs

Depictions of the hidden curriculum were common to medical dramas, most notably representations of hierarchy, unprofessionalism, and patient dehumanization. *Grey’s Anatomy* and *ER* had the highest number of excellent and moderate examples of the hidden curriculum, which may speak to the dramatic nature of these programs. Both television shows demonstrate clear examples of the hidden curriculum, without overwhelming associated plot or character development. We suggest that between these two programs, *ER* may be most suitable as an educational tool. One of the reasons is that *Grey’s Anatomy* tended focus on long-term plot and character development; at times storylines were resolved over a number of episodes, rendering their use for teaching more challenging. As an example, Season 5 focused on one particular character’s personal evolving disease, diagnosis and management, which had little relevance to the hidden curriculum.

Scrubs had the lowest number of examples of the hidden curriculum, possibly due to its comedic nature. In previous research, *Scrubs* was deemed a suitable source of examples to inform medical education in the following spheres: “teaching and learning, mentorship, professionalism, communication skills, and interprofessional relationships” (p. 238, [19]). Our findings, however, challenge the quality of such examples in the context of the hidden curriculum, due to the show’s highly comedic nature. For instance, in episode 10 of Scrubs, the protagonist comments on his view of working females in his profession, and while the message delivered is positive, the comedic scene associated with it diverts the viewer’s attention from the underlying message. As such, in the context of the hidden curriculum we found that this television program was least representative of our focus.

Previous literature has supported the use of television programs to enrich medical education. Hirt and colleagues, systematically analyzed 177 episodes from eight popular medical programs, three of which included *Grey’s Anatomy*, *Scrubs*, and *ER* for their potential use in medical education in academic settings [19]. *Grey’s Anatomy* was shown to have numerous applications in areas related to “hospital environments, communication skills, teaching and learning, ethics, professionalism, and interpersonal conflict” (p. 238, [19]). Interestingly, the authors stated that this television show in particular includes numerous interactions among health professionals of various levels, with a “learn-by-humiliation approach” (p. 240, [19]). Our findings regarding hierarchy, unprofessionalism, and issues of life balance support this previous work, and reveal its applicability for teaching purposes specific to the hidden curriculum as well.

If we consider *ER*, Hirt and colleagues stated that issues related to work-life balance were heavily represented in this medical television drama [19]. Additionally, there was a focus on inter-professional relationships and informed consent. Our findings indicate several such instances which, as per our identified themes, either fell under hierarchy or unprofessionalism. Furthermore, the themes we identified specific to the hidden curriculum relate to Hirt’s findings of *ER* being useful in describing “professionalism, communication skills, ethical issues, inter-professional relationships, procedures, and hospital environments” (p. 238, [19]).

Our findings expand upon another study which examined the frequency of professionalism issues portrayed in the television dramas *Grey’s Anatomy* and *House M.D*. [1]. Czarny and colleagues found a deficiency in the number of commendable portrayals of professionalism, in contrast to a much higher incidence of professionalism breaches, among the two studied medical dramas [1]. Our research uncovered several examples of unprofessionalism regarding truth disclosure, ethical concerns in practice and quality of life issues. In contrast, it was rare for us to find positive examples of professionalism and professional conduct. This may be due in part to the nature of the television shows themselves and dramatic techniques employed to enhance viewer interest.

### Limitations

There are several limitations that merit consideration. One cannot rule out the possibility that the depictions collected in these three television shows may not be a true representation of North American healthcare, and thus not reflect a true hidden curriculum that parallels one in actual medical institutions. We argue that such an inherent limitation merits consideration, despite overlap of similar themes identified in other studies examining the hidden curriculum among medical students [12, 13].

This study considered three television programs out of a multitude of medical dramas. As such, we cannot dismiss the possibility that we would have found other themes if other programs were examined. In addition, only one season was considered for each television program. While we could have viewed more programs, we reached a saturation of themes with the 70 viewed episodes. The selection of season to view may also predispose to an inherent bias. For instance, season five of *Grey’s Anatomy* included a major character plot arc, in which one resident was diagnosed with a life-threatening illness. This resulted in many dramatic scenes centered on personal relationships, rather than professional clinical interactions. Furthermore, as described earlier, *Scrubs* had a surprisingly lower number of depictions of the hidden curriculum. While this may be due to its comedic nature, it may also have been due to the fact that each episode averaged at a running time of 20 min, while *Grey’s Anatomy* was 40 min, and *ER* was 50 min.

### Relevance to education

The examples of the hidden and informal curriculum uncovered in our study have direct relevance to medical education -- the clinical teacher is encouraged to use these examples (see Additional file 1) to address specific themes of the hidden and informal curriculum that they wish students to reflect on during undergraduate and postgraduate teaching.

Ornelas and Parikh [26] have argued that because of negative portrayals of physicians in medical dramas – specifically unethical and unprofessional behaviors – television shows should not be used for educational purposes. We would argue, however, that the fact that the programs depict fictional characters engaging in these behaviors makes them a perfect tool for education. The fact that they are fictional representations allows for trainees to reflect and have open discussions on the behaviors related to the hidden curriculum that are observed, without the worry that they are “passing judgment” on actual individuals.

The behaviors identified during the television shows can also provide the stimulus for students to contrast this behavior with the behavior they witness in clinical practice. Essentially, they can reflect on the question “in the era of Grey’s anatomy (E.R, Scrubs, etc.), are ‘real doctors’ really better than the ones depicted on television?” Are their own behaviors being affected/modeled by what they are watching on television? For example is it possible that their conceptualization of behavior in the clinical domain may in fact be being learned, during the pre-clerkship years, from their television watching?

Although our study did not examine the average viewers’ impressions of the hidden curriculum in medicine, we feel this would be an important area of future study. For example, does the average viewer recognize the conflicts that trainees feel when the formal and informal/hidden curriculum collide? Do they feel these are true depictions of medicine or are simply dramatizations. Studies of viewer perceptions of the hidden curriculum could enhance our knowledge of exactly how patients view the doctors responsible for their care.

## Conclusions

The hidden curriculum is readily depicted in popular medical television shows. Our efforts demonstrate that these examples of the hidden curriculum may serve as a valuable teaching resource in formal undergraduate medical programs, and that these programs can be used to engage students. Specifically, we envision that a database of video vignettes using scenes from these programs could be developed to help in illustrating the hidden curriculum to undergraduate medical students.

## Additional file

Additional file 1: **Emergent coding scheme and hidden curriculum examples by series, episode and time.** (DOC 94 kb)
