# Supplementary material for: Life imitating art: Depictions of the hidden curriculum in medical television programs
Source: BMC Med Educ. 2015 Sep 26;15:156. doi: 10.1186/s12909-015-0437-8 (PMC4583760; doi:10.1186/s12909-015-0437-8)
Supplement: Additional file 1: — Emergent coding scheme and hidden curriculum examples by series, episode and time. (DOC 94 kb) [file 12909_2015_437_MOESM1_ESM.doc]

**Additional file 1**

**Appendix 1**

***Emergent Coding Scheme—Hidden Curriculum TV***

**Consequences of the hidden curriculum.** This refers to the loss of idealism among medical students as they progress from first year into their residency and beyond.

Examples of this include:

-Loss of idealism of trainees or staff -Adoption of a “ritualized” professional identity

-Emotional neutralization/suppression -Change in ethical integrity

**Role Modeling**. Another key element of the hidden curriculum has to do with role modeling, that is, how medical students “model” exemplary behaviour of staff docs.

Examples of this include:

-Trainees modeling/tailoring behaviours to meet the needs of staff docs

-Discussions regarding behaviours they ‘have to model’ but will not continue when they are more independent.

**Unprofessionalism**. This refers to medical students experiencing unprofessionalism.

Examples of:

**-**Unprofessional behaviour from staff physicians.

-Evidence of the tolerance of unprofessional behaviour.

**Hierarchical nature of medicine (disciplines)**. This centers around the primacy put on certain specialties and sub-specialties, and the associated doctors who fill those roles.

Examples of:

-Evidence of primacy

-The perceived “superiority” of some specialties over others

**Hierarchical nature of medicine (position in the hierarchy—inferior/superior)**

-Reinforcement of Junior-Senior positions during interprofessional relations/communication

-Reinforcement of Junior-Senior positions within the medical hierarchy

**Hierarchical nature of medicine (patient perspective)**

**-**Patients reinforcing the hierarchy

**Staging.** Challenges during transitional stages in medicine

Examples of:

-Differences between what you see in the classroom vs on the wards

**Patient dehumanization**

Example of:

-Treating patients as objects/data sources

-Failure to address contextual issues (i.e. psychosocial, etc)

**“Faking it”**

Example of:

-Overstating capabilities

**Appendix 2**

**Table 1a. *Grey’s Anatomy* Hidden Curriculum Examples by Series, Episode and Time**

| Episode | Running time | HC example |
| --- | --- | --- |
| 1 | 3:30-3:33; 4:55-5:09 | Hierarchical nature- discipline |
| 1 | 4:2- 5:49 | Unprofessionalism |
| 1 | 11:40-12:00 | Hierarchical nature: patient point of view |
| 1 | 13:20-13:40 | Hierarchical nature: position |
| 1 | 17:26-17:35 | Hierarchical nature: position |
| 1 | 24:20- 25:16 | Patient dehumanization |
| 2 | 5:10-5:18 | Hierarchical nature: patient point of view |
| 2 | 17:18-17:21 | Hierarchical nature: position |
| 3 | 9:44- 10:31 | Unprofessionalism |
| 3 | 11:35-11:43; | Hierarchical nature: position |
| 3 | 23:38-24:25 | Role modeling |
| 4 | 5:04-5:51 | Hierarchical nature: discipline |
| 4 | 7:20-7:56 | Consequence of HC: change in ethical integrity |
| 4 | 19:20; 22:15; 24:11; | Hierarchical nature: discipline |
| 5 | 08:21-08:30 | Hierarchical nature: discipline |
| 5 | 09:15-09:27; 19:30-20:46 | Unprofessionalism |
| 5 | 11:20-12:14 | Hierarchical nature: position |
| 5 | 16:07-17:31; 30:22-31:30 | Unprofessionalism |
| 6 | 17:26-17:51 | Consequences of the HC |
| 6 | 21:49-22:36; 23:10-24:05 | Staging |
| 6 | 22:36-23:09 | Hierarchical nature: discipline |
| 7 | 28:49-30:21 | Patient dehumanization |
| 10 | 40:46-41:08 | Unprofessionalism |
| 11 | 32:15-33:08 | Hierarchical nature: position |
| 14 | 6:46- 7:54 | Hierarchical nature: patient point of view |
| 14 | 8:56-9:21-12:45 | Role modeling/ patient dehumanization |
| 16 | 30:45-32:10 | Unprofessionalism |
| 17 | 8:06-8:17 | Hierarchical nature: position |
| 18 | 6:28-7:15 | Unprofessionalism |
| 19 | 6:40-7:52 | Hierarchical nature: discipline |
| 19 | 9:56-10:30 | Hierarchical nature: position |
| 20 | 10:10-11:03; 24:36-25:46 | Faking it |
| 22 | 18:25-18:43 | Hierarchical nature: position |
| 23 | 2:36-3:14; | Staging |
| 23 | 3:20-3:59 | Consequence of the HC |
| 23 | 9:55-10:43 | Hierarchical nature: discipline |
| 24 | 1:45-2:31; 17:34-18:22 |  |
| 24 | 9:40-10:31 | Patient dehumanization |
| 24 | 9:56-10:31 | Role modeling |

**Table 1b. *ER* Hidden Curriculum Examples by Series, Episode and Time**

| Episode | Running Time | HC example |
| --- | --- | --- |
| 1 | 5:53-6:02 | Hierarchical nature: position |
| 1 | 6:08-7:00 | Life balance |
| 1 | 11:06-11:52 | Unprofessionalism |
| 1 | 25:35-26:06; 40:03-41:12 | Unprofessionalism |
| 1 | 27:48-28:13 | Life balance |
| 2 | 20:52-21:06 | Hierarchical nature: discipline |
| 2 | 21:39-22:14 | Patient dehumanization |
| 3 | 3:00-3:07 | Consequences of the HC |
| 3 | 6:15-6:28 | Life balance |
| 3 | 17:00-17:08 | Patient dehumanization |
| 3 | 23:24-23:50; 27:58-28:22 | Hierarchical nature: discipline |
| 4 | 7:57-8:47; 13:02-13:36 | Unprofessionalism: misguiding patients |
| 5 | 5:32-6:14 | Hierarchical nature: position |
| 5 | 24:09-24:43 | Hierarchical nature: discipline |
| 6 | 1:02-1:30; 2:23-2:34 | Patient dehumanization |
| 6 | 2:34-2:44 | Hierarchical nature: position |
| 6 | 5:40-6:00 | Life balance |
| 6 | 19:21-2-"13 | Patient dehumanization |
| 9 | 41:05-41:18 | Hierarchical nature: position |
| 10 | 35:09-35:25 | Hierarchical nature: discipline |
| 10 | 37:48-38:06 | Patient dehumanization |
| 11 | 25:05-26:19 | Unprofessionalism |
| 11 | 30:28-30:51 | Hierarchical nature: discipline |
| 12 | 20:17-20:49 | Unprofessionalism |
| 14 | 15:44-17:56 | Unprofessionalism |
| 16 | 12:11-13:25 | Unprofessionalism |
| 17 | 6:30-6:39 | Patient dehumanization |
| 17 | 8:46-9:15 | Unprofessionalism: sexism |
| 21 | 4:53-5:17 | Faking it |

**Table 1c. Scrubs hidden curriculum examples by series, episode and time**

| Episode | Running time | HC example |
| --- | --- | --- |
| 1 | 00:00-00:13 | Unprofessionalism |
| 1 | 12:45-13:16 | Unprofessionalism |
| 2 | 3:20-3:36; 9:51-10:11 | Unprofessionalism; hierarchical nature: position |
| 2 | 6:21-6:50 | Hierarchical nature: position |
| 2 | 12:50-13:14 | Hierarchical nature: position |
| 3 | 3:35-3:44 | Patient dehumanization |
| 4 | 1:05-2:02 | Role Modeling |
| 10 | 1:59- 3:06 | Consequences of the HC |
| 10 | 7:18-7:58; 9:26-10:02 | Hierarchical nature: patient point of view |
| 11 | 2:46-3:11 | Unprofessionalism |
| 12 | 5:54-6:15 | Unprofessionalism |
| 12 | 10:44-11:21 | Hierarchical nature: discipline |
| 12 | 13:35-13:55 | Hierarchical nature: discipline |
| 13 | 1:40-2:09 | Role modeling |
| 13 | 8:18-8:36 | Patient dehumanization |
| 13 | 9:19-9:30 | Hierarchical nature: discipline |
| 15 | 6:29-6:50 | Hierarchical nature: patient point of view |
| 15 | 8:24-8:40 | Patient dehumanization |
| 15 | 8:24-8:46 | Hierarchical nature: position |
| 15 | 11:28-11:54 | Unprofessionalism |
| 16 | 5:54-6:16 | Hierarchical nature: discipline |
| 20 | 3:41-3:55 | Hierarchical nature: discipline |
| 24 | 3:11-5:12 | Unprofessionalism |
| 24 | 14:34:15:13 | Patient dehumanization |
| 25 | 5:13-5:35 | Unprofessionalism |
